# SGLT‐1‐specific inhibition ameliorates renal failure and alters the gut microbial community in mice with adenine‐induced renal failure

**DOI:** 10.14814/phy2.15092

**Published:** 2021-12-18

**Authors:** Hsin‐Jung Ho, Koichi Kikuchi, Daiki Oikawa, Shun Watanabe, Yoshitomi Kanemitsu, Daisuke Saigusa, Ryota Kujirai, Wakako Ikeda‐Ohtsubo, Mariko Ichijo, Yukako Akiyama, Yuichi Aoki, Eikan Mishima, Yoshiaki Ogata, Yoshitsugu Oikawa, Tetsuro Matsuhashi, Takafumi Toyohara, Chitose Suzuki, Takehiro Suzuki, Nariyasu Mano, Yoshiteru Kagawa, Yuji Owada, Takane Katayama, Toru Nakayama, Yoshihisa Tomioka, Takaaki Abe

**Affiliations:** ^1^ Department of Medical Science Tohoku University Graduate School of Biomedical Engineering Sendai Japan; ^2^ Division of Nephrology, Endocrinology and Vascular Medicine Tohoku University Graduate School of Medicine Sendai Japan; ^3^ Department of Medical Megabank Tohoku University Sendai Japan; ^4^ Department of Biomolecular Engineering Applied Life Chemistry Tohoku University Graduate School of Engineering Sendai Japan; ^5^ Department of Clinical Biology and Hormonal Regulation Tohoku University Graduate School of Medicine Sendai Japan; ^6^ Department of Pharmaceutical Sciences Tohoku University Hospital Sendai Japan; ^7^ Department of Integrative Genomics, Tohoku Medical Megabank Organization Tohoku University Sendai Japan; ^8^ Laboratory of Oncology Pharmacy Practice and Sciences Tohoku University Graduate School of Pharmaceutical Sciences Sendai Japan; ^9^ Laboratory of Animal Products Chemistry Graduate School of Agricultural Science Tohoku University Sendai Japan; ^10^ Department of Pediatrics Tohoku University Graduate School of Medicine Sendai Japan; ^11^ Department of Organ Anatomy Tohoku University Graduate School of Medicine Sendai Japan; ^12^ Laboratory of Molecular Biology of Bioresponse Graduate School of Biostudies Kyoto University Kyoto Japan

**Keywords:** chronic kidney disease, gut microbiota, phenyl sulfate, sodium glucose cotransporter 1 (SGLT1), uremic toxins

## Abstract

Sodium‐dependent glucose cotransporters (SGLTs) have attracted considerable attention as new targets for type 2 diabetes mellitus. In the kidney, SGLT2 is the major glucose uptake transporter in the proximal tubules, and inhibition of SGLT2 in the proximal tubules shows renoprotective effects. On the other hand, SGLT1 plays a role in glucose absorption from the gastrointestinal tract, and the relationship between SGLT1 inhibition in the gut and renal function remains unclear. Here, we examined the effect of SGL5213, a novel and potent intestinal SGLT1 inhibitor, in a renal failure (RF) model. SGL5213 improved renal function and reduced gut‐derived uremic toxins (phenyl sulfate and trimethylamine‐*N*‐oxide) in an adenine‐induced RF model. Histological analysis revealed that SGL5213 ameliorated renal fibrosis and inflammation. SGL5213 also reduced gut inflammation and fibrosis in the ileum, which is a primary target of SGL5213. Examination of the gut microbiota community revealed that the Firmicutes/Bacteroidetes ratio, which suggests gut dysbiosis, was increased in RF and SGL5213 rebalanced the ratio by increasing Bacteroidetes and reducing Firmicutes. At the genus level, *Allobaculum* (a major component of Erysipelotrichaceae) was significantly increased in the RF group, and this increase was canceled by SGL5213. We also measured the effect of SGL5213 on bacterial phenol‐producing enzymes that catalyze tyrosine into phenol, following the reduction of phenyl sulfate, which is a novel marker and a therapeutic target for diabetic kidney disease DKD. We found that the enzyme inhibition was less potent, suggesting that the change in the microbial community and the reduction of uremic toxins may be related to the renoprotective effect of SGL5213. Because SGL5213 is a low‐absorbable SGLT1 inhibitor, these data suggest that the gastrointestinal inhibition of SGLT1 is also a target for chronic kidney diseases.

## Graphical abstract

Inhibition of gut SGLT1 by a compound SGL5213 in adenine‐induced renal failure mice ameliorated renal dysfunction and renal fibrosis and inflammation. Restoring gut microbiota community was also found. These data suggest that the gastrointestinal inhibition of SGLT1 is a novel target for chronic kidney diseases.

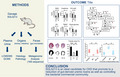

## 1 INTRODUCTION

Chronic kidney disease (CKD) is a worldwide health problem, and its complications increase the risk of progression to end‐stage renal disease (ESRD), cardiovascular disease, and all‐cause mortality (Jha et al., 2013; Weiner et al., 2004). Diabetic kidney disease (DKD) occurs in approximately 20–30% of all diabetic patients and is a major cause of ESRD, cardiovascular events, and death (Ritz & Orth, 1999). Recently, sodium‐dependent glucose cotransporters (SGLTs) have attracted considerable attention as new targets for type 2 diabetes (T2DM) (Alicic et al., 2019). In the kidney, SGLT2 is responsible for reabsorption of more than 90% of filtered glucose, and SGLT1 reabsorbs the remaining 10% (Kalra et al., 2016; Wright et al., 2011). Several SGLT2 inhibitors have been approved for the treatment of T2DM, and it has been reported that the SGLT2‐specific inhibitors empagliflozin (Wanner et al., 2016) and canagliflozin (Perkovic et al., 2018) are associated with slower progression of kidney disease, suggesting a possible renoprotective effect in T2DM.

In contrast, SGLT1 plays an important role in glucose absorption in the small intestine (Wright et al., 2011). Intestinal SGLT1 mRNA expression and glucose uptake are increased in patients with T2DM (Kuroda et al., 2019). Therefore, SGLT1 inhibitors are an attractive option for the treatment of T2DM (Kuroda et al., 2019). In addition, recent studies have revealed that inhibition of SGLT1 in the small intestine results in reduced glucose uptake and that unabsorbed glucose transiently reaches the lower small intestine (Oguma et al., 2015; Roder et al., 2014), which led to the conjecture that the glucose load may alter the gut microbiota composition and reduce gut‐derived uremic toxins. Indeed, we previously reported that the dual sodium/glucose co‐transporter (SGLT) 1/2 inhibitor canagliflozin reduced the accumulation of gut‐derived uremic toxins, indoxyl sulfate (IS), and *p*‐cresyl sulfate (PCS), by altering the intestinal environment in an adenine‐induced renal failure (RF) mouse model (Mishima et al., 2018) However, the precise relationship between SGLT1‐specific inhibition and renal function as well as gut‐derived uremic toxins is still unclear. Here, we examined the effect of a low‐absorbable SGLT1 inhibitor, SGL5213 (Io et al., 2019; Kuroda et al., 2019), on renal function, uremic toxins, and gut microbiota in an adenine‐induced RF model.

## 2 MATERIALS AND METHODS

### 2.1 Materials

SGL5213, a low‐absorbed SGLT1 inhibitor (Io et al., 2019; Kuroda et al., 2019) was supplied by Taisho Pharmaceutical Co., Ltd. Adenine was purchased from Wako Pure Chemical Industries, and a sterilized adenine‐containing diet (0.2%) was purchased from CLEA Japan. S‐(*o*‐nitrophenyl)‐L‐cysteine (SOPC) was synthesized according to the method described by Phillips et al. (1989), in which 2‐nitrofluorobenzene was replaced with 1,2‐dinitrobenzene.

### 2.2 Animal model

All experiments were approved in accordance with the guidelines of the Animal Ethics Committee of Tohoku University School of Medicine. C57BL/6N mice were purchased from CLEA Japan Inc. Seven‐week old mice were used for the intestinal glucose transit experiment, as well as for the 2 week administration of SGL5213 in the normal and adenine‐induced RF models.

For the experiment of intestinal glucose experiment, normal (Nor), and adenine‐induced RF mice were administered a 10 mg/kg glucose with or without bolus of 100 mg/kg SGL5213 or vehicle (dH_2_O) in a 100 μl volume after 20–22 h of fasting.

After 1 h, the mice were sacrificed, and blood, urine, feces, and tissues were collected.

To generate the RF model, the mice were fed a CE‐2 diet containing 0.2% adenine (0.2% adenine diet) for at least 6 weeks (Mishima et al., 2015). After that, the 0.2% adenine diet was subsequently fed for an additional week and then changed to the normal diet for another week, as previously reported (Kamijo‐Ikemori et al., 2016). During these 2 weeks, the mice were orally administered 10 mg/kg SGL5213 (dissolved in water) twice per day. Water intake and food intake were estimated by calculating the total water intake in one group cage (divided per mouse number). At the end of the study, the mice were sacrificed after isoflurane anesthesia, and blood, urine, feces, kidney, intestine, cecum, and colon tissues were collected. Blood urea nitrogen and biochemical parameters were assessed using a blood analyzer (i‐STAT; Fuso Pharmaceutical Industries). The plasma, urine, feces, and intestinal glucose levels of the control and RF mice were measured using LabAssay™ Glucose (Mutarotase‐GOD method, Wako) according to the manufacturer's instructions. Mouse tail blood pressure was measured as we previously described (Nanto‐Hara et al., 2020).

### 2.3 Histological analysis

Tissues were fixed in 10% neutral‐buffered formalin and embedded in paraffin. Kidney sections were stained with hematoxylin and eosin (H&E) and Masson's trichrome (MT) staining (Mishima et al., 2015; Nanto‐Hara et al., 2020). The renal tubular areas in the cortex were quantitatively analyzed using MT‐stained sections using Image J analysis software (National Institutes of Health). Two pathologists, who were blinded to the treatment of the individual mice, performed the evaluation.

### 2.4 Quantitative polymerase chain reaction

Tissue samples were homogenized in TRIzol reagent (Invitrogen, Thermo Fisher Scientific), and mRNA was extracted according to the manufacturer's instructions. cDNA synthesis was performed using a Transcriptor First Strand cDNA Synthesis Kit (Roche). Primers were purchased from Applied Bio systems/Thermo Fisher Scientific (Table S1).

### 2.5 Measurement of uremic toxins

Plasma concentrations of TMAO, phenyl sulfate (PS), and IS were measured using liquid chromatography–tandem mass spectrometry (LC‐MS/MS), as described previously (Kanemitsu et al., 2017). Briefly, chromatographic separation was performed on a Nanospace SI‐2 LC system (Shiseido) using a Scherzo SS‐C18 analytical column (50 ×  2.0 mm i.d., 3.0 µm, Imtakt). A guard column (2 ×  5 mm, 3 μm) was used to protect the analytical column containing the same material as the analytical column, which was fitted between the analytical column and the autosampler. The column effluent was monitored using a TSQ Ultra triple quadrupole mass spectrometer (Thermo Fisher Scientific) equipped with a heated electrospray ionization source system. Samples were analyzed in the single reaction monitoring mode using the ion transitions: *m*/*z* 76.05→58.10 for TMAO, *m*/*z* 172.99→93.30 for PS, and *m*/*z* 212.03→131.95 for IS. Deuterated internal standards were used for all analytes.

### 2.6 Microbiome analysis

Genomic DNA of gut microbiota was extracted from the murine fecal contents, and 16S rRNA genes in the DNA samples were analyzed using a MiSeq sequencer (Illumina) as described previously (Murakami et al., 2015) by Bioengineering Lab. Co. Ltd.. Briefly, microbial genomic DNA was extracted using a phenol‐chloroform standard protocol with vigorous shaking with 0.1‐mm zirconia/silica beads. The V1‐V2 region of the 16S rRNA gene was amplified from the isolated DNA using the bacterial universal primer set 27Fmod (5′‐ACACTCTTTCCCTACACGACGCTCTTCCGATCTAGRGTTTGATYMTGGCTCAG‐3′) and 338R (5′‐GTGACTGGAGTTCAGACGTGTGCTCTTCCGATCTTGCTGCCTCCCGTAGGAGT‐3′). MiSeq sequencing was performed according to the manufacturer's instructions. The 16s rRNA reads were analyzed using QIIME (v1.9.1) (Caporaso et al., 2010). Microbiome data were deposited in the DDBJ database (Accession # DRA011963).

### 2.7 TPL enzyme activity

The TPL enzyme of *Erwinia herbicola* was purified as previously reported (Katayama et al., 2000). Enzyme activity was measured with 0.6 mM SOPC (Watkins & Phillips, 2001) in 50 mM potassium phosphate, pH 8.0, at 25°C, following the decrease in absorbance at 370 nm (¢ = −1.86 × 10^3^ M^−1^ cm^−1^) (Phillips, 1987). The inhibitory effects of 2‐Aza tyrosine (Kikuchi et al., 2019; Watkins & Phillips, 2001) and SLG5213 on TPL were determined using SOPC as the substrate, as described above. The reaction mixtures contained 50 mM triethanolamine hydrochloride, pH 6.8–8.8, or 50 mM Bis‐tris propane hydrochloride, pH 8.3–9.4, 2.5 mM dithiothreitol, 50 μM PLP, 0.2 mM NADH, 2 units of LDH, and L‐tyrosine, and the reaction was initiated by the addition of TPL (Sundararaju et al., 1997).

### 2.8 Statistical analysis

Results are presented as mean ± SE. The data of the Chao 1 and Shannon index are presented as box plots. To compare two groups, we used Student‐*t* test. Comparisons in three group were assessed using a Dunnett's test with JMP Pro software version 13 (SAS Institute Inc.). Statistical significance was set at a *p* value <0.05.

## 3 RESULTS

### 3.1 Effects of the SGLT1‐selective inhibitor SGL5213 in normal and RF mice

**FIGURE 1:**
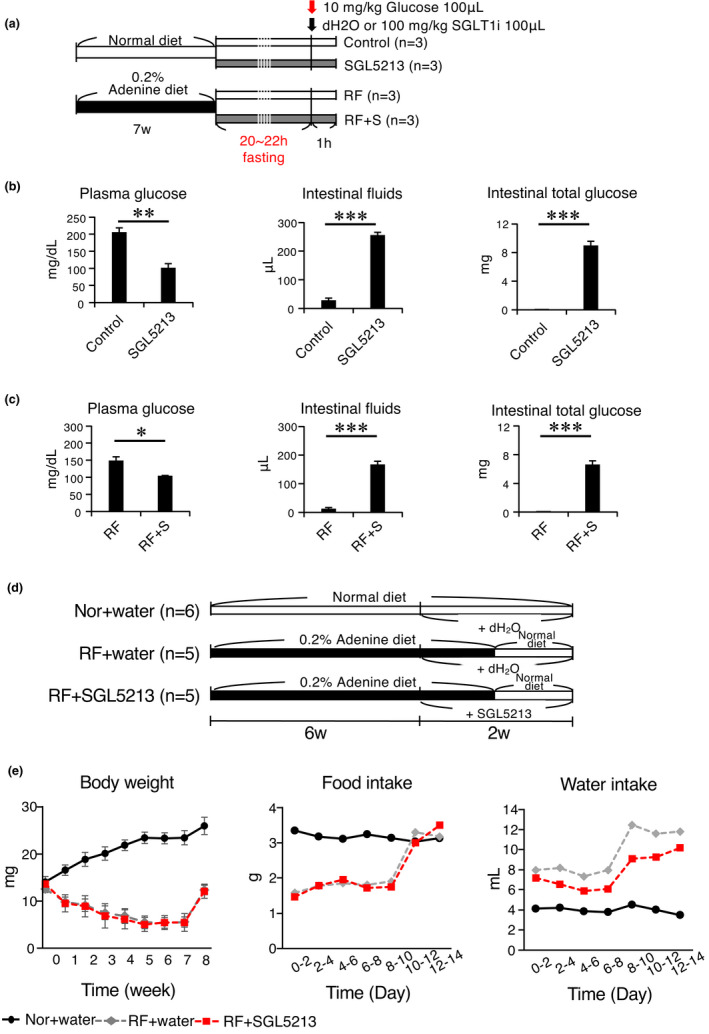
Experimental design and effect of SGL5213 on gut fluid and glucose of normal and renal failure mice. (a) Mice were divided into four subgroups: (1) a control group (*Control*), (2) an SDL5213 (100 mg/kg)‐bolus treated control group (SGL5213), (3) an adenine‐induced renal failure group (*RF*), and (4) an SGL5213 (100 mg/kg)‐bolus treated RF group (*RF* + S). Mice were administered a 10 mg/kg glucose with or without bolus of 100 mg/kg SGL5213 or vehicle (dH2O) in a 100 μl volume after 20–22 h of fasting. (b) Plasma glucose, intestinal fluid, and intestinal total glucose in normal mice. Control: normal group (*n* = 8); SGL5213: Normal diet treated with SGL5213 (100 mg/kg/day) group (*n* = 8–9 each). Statistical analyses were performed using a Student‐*t* test. *p < 0.05, ***p < 0.01, and ***p < 0.01 were treated as statistically significant. (c) Plasma glucose, intestinal fluid, and intestinal total glucose in renal failure mice. RF: renal failure group (*n* = 8); RF + S: RF treated with SGL5213 (100 mg/kg/day) group (*n* = 8–9 each). Data were mean ± SEM. Data were mean ± SEM. Statistical analyses were performed using a Student‐*t* test. *p < 0.05, ***p < 0.01, and ***p < 0.01 were treated as statistically significant. (d) Mice were divided into three subgroups: (1) a control normal diet group *(Control*), (2) an adenine‐induced uremic renal failure group (*RF*), and (3) an SGL5213 (10 mg/kg/day)‐treated RF group (*RF* + S). (e) Body weight was measured weekly. Food and water intake were measured every other day

To examine the effect of SGL5213 on the acute postprandial peak glucose concentration, we administered a bolus of SGL5213 and examined the glucose dynamics in normal and RF mice (Figure 1a). In normal mice, the plasma glucose level was reduced by SGL5213; in the small intestine, SGL5213 increased the intestinal fluid and the unabsorbed amount of intestinal glucose, resulting in an increase in the total amount of intestinal glucose (Figure 1b). Under RF conditions, SGL5213 also reduced blood glucose levels and increased both intestinal fluid and total intestinal glucose levels (Figure 1c). These results suggest that SGL5213 inhibits intestinal glucose uptake during RF.

### 3.2 Effect of SGL5213 on adenine‐induced RF mice

Next, we examined the effect of long‐term administration of SGL5213 in the RF model (Table 1, Figure 1d). Body weight and food intake were reduced, and water intake was increased in the adenine groups (Figure 1e). After changing to the normal diet for another week, body weight, and food intake increased. In the RF‐SGL5213 group, water intake was reduced, and Hb and Hct were increased compared with the RF group, suggesting an improvement in renal function (Table 1). We also measured heart rate and blood pressure in normal, RF, and RF + SGL5213 mice, but there were no significant differences in heart rate, systolic blood pressure, and mean blood pressure. Only diastolic blood pressure in RF mice was lower than that in normal mice but we did not observe any significant meaning in it. This suggests that the renoprotective effect of SGL5213 was not dependent on blood pressure (Figure S1).

**TABLE 1:** Water and food intake, plasma glucose levels, and biochemical parameters in adenine‐induced renal failure model

	Control (*n* = 6)	RF (*n* = 5)	RF + S (*n* = 5)
Na (mmol/L)	147.5 ± 0.8	145.4 ± 1.1	145.6 ± 0.5
K (mmol/L)	5.2 ± 0.2	5.1 ± 0.3	5.2 ± 0.3
Cl (mmol/L)	109.7 ± 0.4	108.6 ± 0.9	108.8 ± 1.0
tCO_2_ (mmol/L)	23.0 ± 0.5	23.4 ± 0.9	23.6 ± 0.2
Hb (mg/dL)	15.7 ± 0.2***	10.2 ± 0.2	11.2 ± 0.3*
Hct (%PCV)	46.2 ± 0.7***	30.0 ± 0.6	33.0 ± 0.8*
AnGap (%PCV)	21.0 ± 0.6	19.2 ± 0.9	19.0 ± 0.8

Note

Body weight, food intake, water intake, and biochemical parameters (Na, K, Cl, tCO_2_, total carbon dioxide; Ht, hematocrit; and Hb, hemoglobin) were measured. Statistical analysis was performed using a Dunnett's test compared with the RF group. *p < 0.05 and ***p < 0.01 were treated as statistically significant. RF; renal failure, RF + S; and renal failure + SGL5213 (10 mg/kg).

Mean ± SEM, *n* = 5–6, *p < 0.05, ***p < 0.001 versus RF (Dunnett test).

**FIGURE 2:**
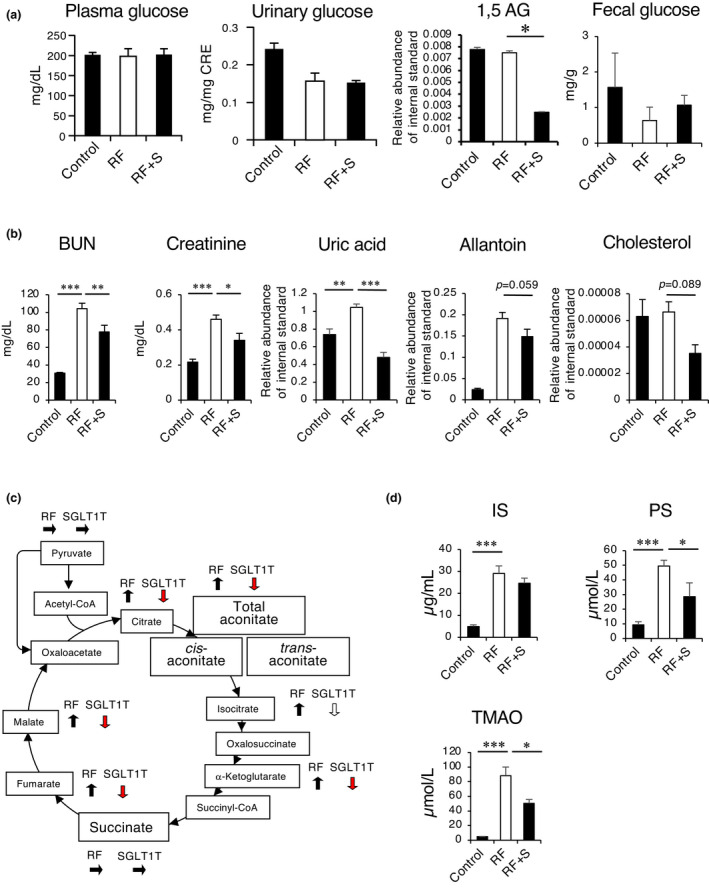
SGL5213 improved renal function in an adenine‐induced renal failure model. (a) Plasma glucose, urine glucose, and 1,5‐AG levels in adenine‐induced renal failure model. (b) The plasma BUN, Cr, Urea, allantoin, and cholesterol levels in adenine‐induced renal failure model mice treated with SGL5213 (10 mg/kg/day) for 14 days. Cont: control group (*n* = 8); RF; renal failure group (*n* = 6); RF treated with SGL5213 (10 mg/kg/day) group (*n* = 8–9 each). (c) The plasma TCA component levels. (d) The plasma level of IS, PS, and TMAO in the adenine‐induced renal failure model. Cont; control group (*n* = 8), RF: renal failure group (*n* = 6), RF treated with SGL5213 (10 mg/kg/day) group (*n* = 8–9 each). Data were mean ± SEM. Statistical analysis was performed using a Dunnett's test. *p < 0.05 and ***p < 0.01 were treated as statistically significant

Next, we performed biochemical analyses of metabolites. Blood and urine glucose levels were not altered by SGL5123 treatment. 1,5‐anhydroglucitol (1,5‐AG), a nonmetabolizable glucose analog that competes with glucose for renal reabsorption (Balis et al., 2014), was reduced (Figure 2a). Although the bioavailability of SGL5213 was only 0.17% (Kuroda et al., 2018), it suggests that SGL5123 inhibits kidney SGLT1 to some extent without affecting serum or the total amount of urinary glucose.

We also measured fecal glucose and found that there was no significant difference between the levels of the RF and RF + SGL5213 groups (Figure 1), suggesting that utilization of excreted glucose in the intestine by gut microbiota and consumption caused a low level of fecal glucose in the RF + SGL5213 group.

Next, we examined renal function and the metabolite analysis (Figure 2b). In the adenine‐induced RF mice, BUN and Cr levels were increased. Under these conditions, the increased BUN and Cr levels in RF mice were significantly reduced by SGL5213.

Metabolomic analysis revealed that ureic acid was significantly an allantoin was tend to be reduced by SGL5213, further suggesting the improvement of renal function and oxidative stress, as allantoin is a major xanthine product in mice as well as a marker of oxidative stress (Kand'ar & Zakova, 2008). No change of the cholesterol level was seen in this experiment.

In RF, the serum values of citrate, fumarate, oxalate, and malate were significantly increased, so that the sum of the concentrations of the TCA cycle intermediates was increased (Biasioli et al., 1987). Therefore, we measured the levels of the TCA components. As shown in Figure 2c, the serum levels of citrate, isocitrate, α‐ketoglutarate, fumarate, and malate were increased in the RF group, and SGL5213 significantly reduced the aconitic acid, citrate, α‐ketoglutarate, fumarate, and malate levels, further suggesting an improvement in renal function (data also in Figure S2). Because SGL5213 exhibited low membrane permeability and the bioavailability of SGL5213 was very low in vivo, indicating a low absorbability (Kuroda et al., 2019), the main active site of SGL5213 is suggested to be in the gut.

Recently, we reported that canagliflozin, a dual SGLT1/2 inhibitor, reduced the plasma levels of gut‐derived uremic toxins, IS, and *p*‐cresyl sulfate (PCS), by influencing the intestinal environment in mice with adenine‐induced RF (Mishima et al., 2018, 2015). Therefore, we examined the effect of SGL5213 on gut‐derived uremic toxin levels. As shown in Figure 2d, the plasma levels of IS, PS, and TMAO were significantly increased in the RF mice, and the elevated plasma levels of TMAO and PS were significantly reduced in SGL5213 mice, although the elevated level of IS did not change. As we reported, IS, PS, and PCS were 100% gut‐derived. Concerning TMAO, 70% of TMAO was gut derived, and 30% was from TMA in a chaw with fish meal. Therefore, the changes in PS and TMAO were very close to the changes in the gut microbial community (Kikuchi et al., 2019; Mishima, Fukuda, Mukawa, et al., 2017). These results suggest that SGL5213 alters the specific gut microbial community during RF.

**FIGURE 3:**
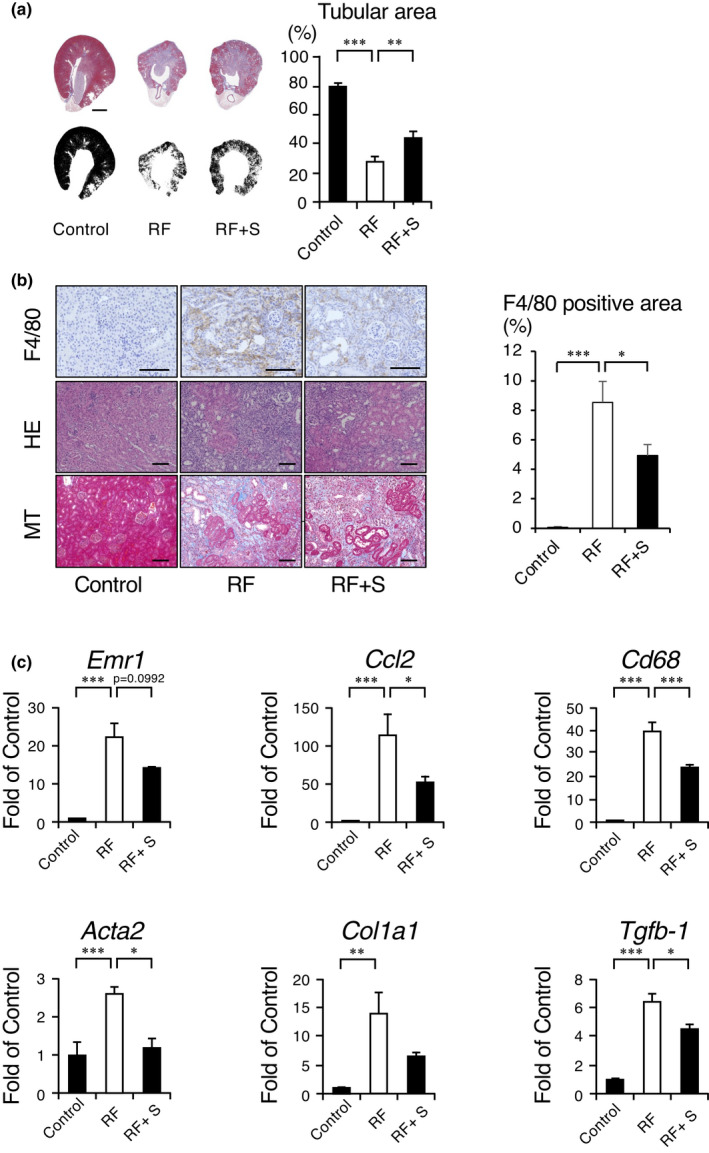
Histological examination of the adenine‐induced renal failure mouse kidney. (a) Masson's trichrome (MT) staining was performed. Morphometric analysis of the cortical tubular area was calculated based on the images. Bars = 500 μm. Data were mean ± SEM. Statistical analysis was performed using a Dunnett's test. *p < 0.05 and ***p < 0.01 were treated as statistically significant. (b) Representative histological images of the adenine‐induced renal failure mouse kidney. Masson's trichrome stained (MTS) and immunohistochemical analysis of F4/80 was also performed. Bars = 50 μm. Arrow indicates the F4/80‐positive macrophages. (c) Quantitative analysis of fibrotic and inflammatory genes. The mRNA levels of *emr1*, *ccl2*, c*d68*, *act2*, *col1a1*, and *tfb*‐*1* were measured by real‐time PCR. The mRNA expression levels were normalized with β‐actin. Data were mean ± SEM. Statistical analysis was performed using a Dunnett's test. *p < 0.05 and ***p < 0.01 were treated as statistically significant

Next, we performed histological analysis of the kidney. The cortical tubular area described by MT staining was decreased in the RF group, and the reduction was significantly recovered by SGL5213 (Figure 3a, left). In addition, the increase in the renal fibrotic area in the RF group was ameliorated by SGL5213. Immunostaining with F4/80 (an antigen that is a macrophage‐restricted cell surface glycoprotein) showed an increase in the fibrotic area in the RF mice, and this increase was reduced by SGL5213.

Quantitative analysis also showed that reduction of fibrotic area (Figure 3a, right panel). These data suggest the recovery of the tissue damaged by SGL5213.

Next, we examined fibrosis‐ and inflammation‐related gene levels by qPCR. The expression levels of fibrosis‐related genes, *acta2* (α‐SMA), *col1a1* (collagen I), and *tgfb1* (TGFβ1), were upregulated in the RF mice, and the upregulated levels of *acta2* and *tgfb1* were significantly decreased by SGL5213 (Figure 3b). The expression levels of inflammation‐related genes, *emr1* (F4/80), *ccl2* (MCP‐1), and *cd68* (CD68) were increased in RF, and the upregulated levels of *ccl2* and *cd68* were significantly decreased by the drug. These data suggest that SGL5213 ameliorated renal damage and reduced fibrosis and macrophage infiltration in an adenine‐induced RF model.

### 3.3 SGL5213 reduced gut inflammation and fibrosis

**FIGURE 4:**
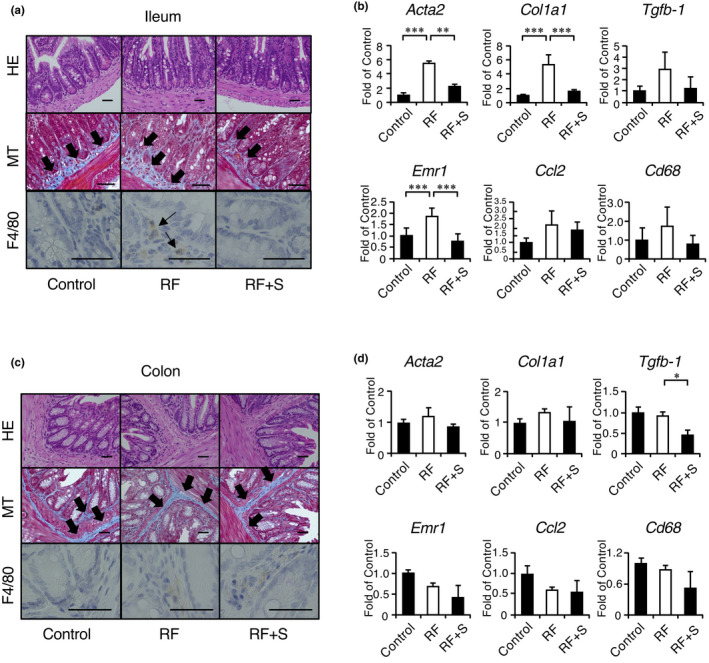
SGL5213 ameliorated intestinal fibrosis and inflammation in the adenine‐induced renal failure model. (a) Representative histological images of the adenine‐induced renal failure mouse ileum. Masson's trichrome (MT) staining was performed. In the MT, the arrow indicates the fibrotic area. In the MTS, the arrow indicates dense eosinophilic granules. Bars = 50 μm. Immunohistochemical analysis of F4/80 was also performed. Arrow indicates the F4/80‐positive macrophages. (b) Quantitative analysis of fibrotic and inflammatory genes in the ileum. The mRNA levels of fibrotic genes *(act2*, *col1*, and *tgfb1*) and inflammatory genes *(emr1*, *ccl2*, and *cd68*) were measured by real‐time PCR. The mRNA expression levels were normalized to that of GAPDH. Data were mean ± SEM. Statistical analyses were performed using a Dunnett's test. *p < 0.05 and ***p < 0.01 were treated as statistically significant. (c) Representative histological images of adenine‐induced renal failure in the mouse colon. SR staining was performed. Bars = 50 μm. Immunohistochemical analysis of F4/80 and claudin 1 was also performed. (d) Quantitative analysis of fibrotic and inflammatory genes in the colon. The mRNA levels of fibrotic genes (*act2*, *col1*, and *tgfb1*) and inflammatory genes (*emr1*, *ccl2*, and *cd68*) were measured by real‐time PCR. The mRNA expression levels were normalized to that of GAPDH. Data were mean ±SEM. Statistical analyses were performed using a Dunnett's test. *p < 0.05 and ***p < 0.01 were treated as statistically significant

It has been reported that disruption of the tight junction in the gastric, ileal, jejunal, and colonic epithelium was observed in RF (Nanto‐Hara et al., 2020; Vaziri et al., 2013). This impairment of barrier function in CKD could contribute to the pathogenesis of systemic inflammation in CKD (Vaziri et al., 2016). We then examined the effect of SGL5213 on the uremic gut in an adenine‐induced RF model. MT staining showed fibrotic changes in the RF group, but the fibrotic areas were decreased by SGL5213 (Figure 4a). Immunostaining with F4/80 also showed an increase in the area of the RF, and this increase was recovered by SGL5213. qPCR analysis revealed that the expression levels of fibrotic genes *acta2* and *col1a1* were significantly increased, but the expression level of *tgfb1* was not increased in the RF group (Figure 4b). The upregulated expression levels of *acta2* and *col1a1* were significantly reduced by SGL5213. In addition, qPCR analysis of the inflammatory genes *emr1*, *ccl2*, and *cd68* also revealed that the gene expression level of F4/80 (*emr1*) was significantly increased in the RF group, and this increase was significantly reduced by SGL5213 treatment (Figure 4b). These data suggest that SGL5213 reduces ileal fibrosis, accompanied by RF.

In contrast, in the colon, neither MT nor F4/80 staining showed significant changes in the RF and RF + SGL5213 groups compared with the control group (Figure 4c). qPCR analysis also revealed that the expression levels of *acta2*, *col1a1*, and *tgfb1* were not changed in the RF groups, and the expression level of *tgfb1* was only decreased in the RF + SGL5213 group compared to the RF group. Furthermore, no significant changes were observed in the inflammatory genes *emr1*, *ccl2*, and *cd68* (Figure 4d). These results suggest that SGL5213 reduces gut inflammation and fibrosis mainly in the ileum following modification of the gut environment.

### 3.4 SGL5213 altered gut‐derived uremic toxins and the gut microbiota community in RF mice

**FIGURE 5:**
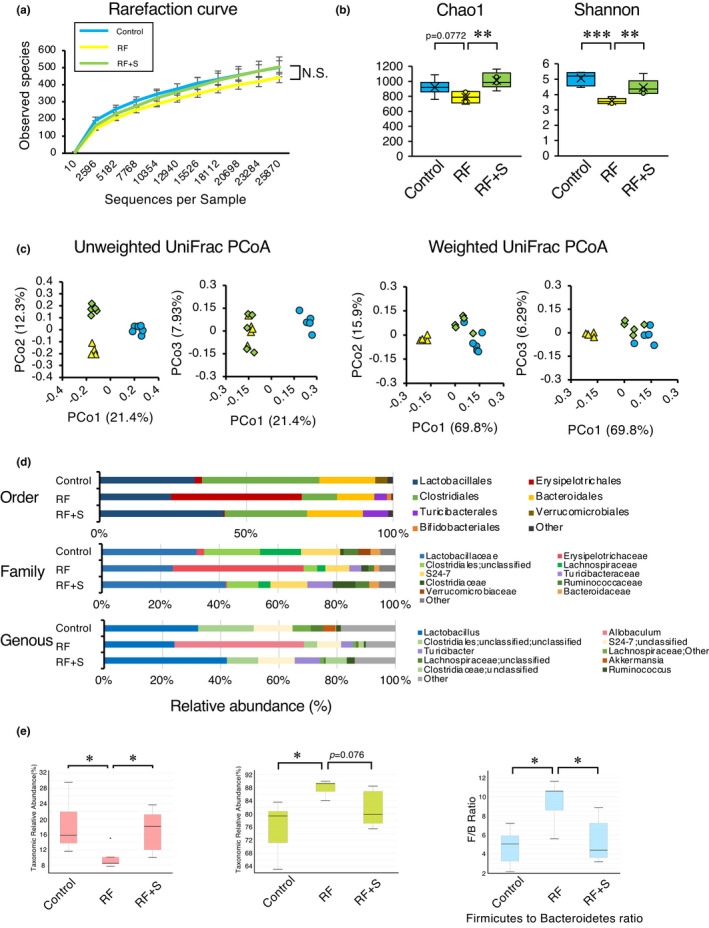
Effect of SGL5213 on the gut‐derived uremic toxins and microbiome community in renal failure mice. (a) Observed OTU rarefaction analysis. OTU rarefaction curves of gut microbiota were used to estimate richness in the control (Cont), renal failure (RF), and RF + SGL5213 groups (10 mg/kg/day). (b) OTU‐based α‐diversity of each microbiome. Significant diversity was seen between control versus RF and RF versus RF + SGL5213 by Chao 1 (left) and Shannon (right) analyses. (c) Principal coordinates analysis of the microbiome profiles using weighted UniFrac. The scores for the first principal component (PC1) versus the second principal component (PC2) and PC1 versus the third principal component (PC3) are presented. (d) Relative abundance of microbiota based on the average abundance of each subgroup at order, family, and genus levels. Major subgroups are indicated on the right. Genera displaying a significant change among the three groups. The *y*‐axis indicates the abundance of each microbe (%). Control (*n* = 8), RF (*n* = 6), RF + SGL5213 (*n* = 8), and RF + SGL5213 (*n* = 9) in each group. Data were mean ± SEM. (e) The Firmicutes/Bacteroidetes ratio (F/B ratio) was calculated as a biomarker of gut dysbiosis. A decrease of Bacteroidetes along with an increase of Firmicutes resulted in a dysbiosis signature of gut microbiota in the renal failure. SGL5213 significantly rebalanced the F/B ratio. Statistical analyses were performed using a Dunnett's test. *p < 0.05 was treated as statistically significant

To elucidate the effect of SGL5213 on the gut microbial community, we performed fecal 16S rRNA gene analysis. As shown in Figure 5a, the rarefaction curve showed no differences in species richness among the groups. However, the Chao 1 index (richness) and Shannon index evenness revealed lower richness and diversity in the RF group, and the reduced Chao 1 and Shannon indices were restored by SGL5213 (Figure 5b). In addition, UniFrac analysis (unweighted, left panel and weighted, right panel) revealed that the clustering patterns of normal, RF, and SGL5213 groups were separated (Figure 5c). These data suggest that SGL5213 affects the gut microbial community during RF.

Next, we focused on the parts of the microbiome in which the abundances were positively correlated with the plasma gut‐derived uremic toxin levels in RF and were changed by SGL5213. Figure 5d shows the microbial population at the phylum, class, order, family, and genus levels. At the phylum level, we found three major populations: Firmicutes, Bacteroidetes, and Actinobacteria. The Firmicutes/Bacteroidetes (F/B) ratio has been widely considered a signature of gut dysbiosis (Yang et al., 2015). As shown in Figure 5e, compared with control mice, the RF mice showed an increased F/B ratio caused by the expansion of Firmicutes and contraction of Bacteroidetes. In addition to the reduced gut‐derived uremic toxins, SGL5213 was able to rebalance the dysbiotic gut microbiota by reducing the F/B ratio by increasing Bacteroidetes but reducing the OTU (operational taxonomic unit) level of Firmicutes. Furthermore, Actinobacteria was increased in the RF group, and this increase was significantly attenuated by SGL5213. These results further suggest that SGL5213 alters the specific gut microbial community during RF.

**FIGURE 6:**
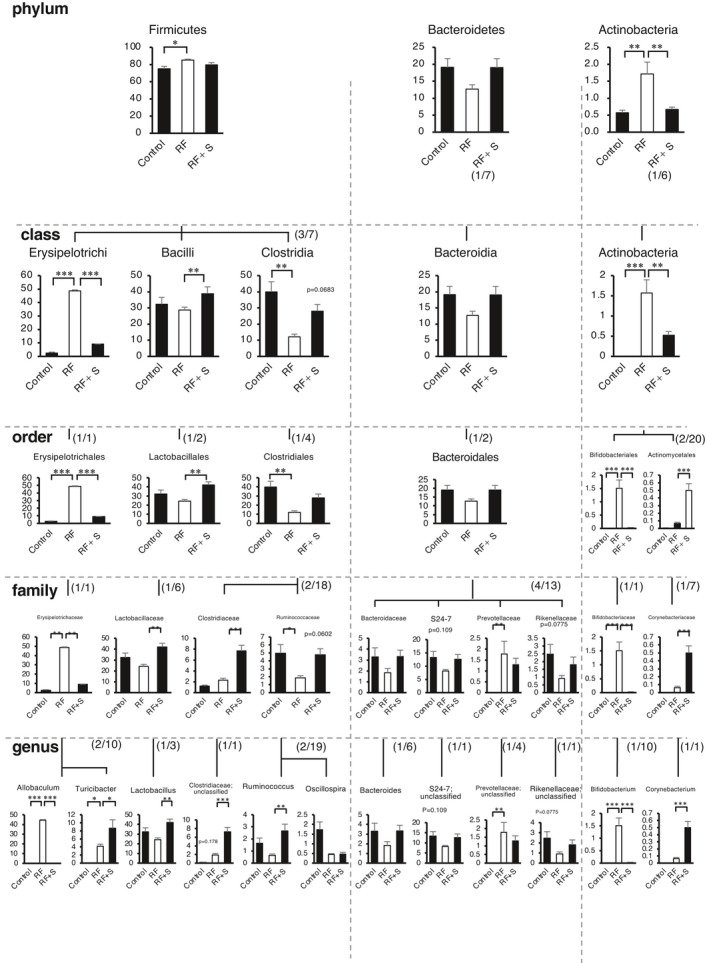
Relative abundance of microbiota based on the average abundance of each subgroup at order, family, and genus levels. Statistical analysis was performed using a Dunnett's test. *p < 0.05 and **p < 0.01 were treated as statistically significant. Cont: control group, RF: renal failure group; RF + SGL5213 (*n* = 8 for each group)

Figure 6 shows the changes in the gut microbial community throughout the bacterial tree diagram. Among these, we next focused on the parts of the microbiome in which the abundance positively correlated with the plasma uremic toxin levels when modified by SGL5213. At the genus level within Firmicutes, the most represented genera in all subjects were *Allobaculum* (a major component of Erysipelotrichaceae). *Allobaculum* was significantly increased in the RF group, but this increase was significantly ameliorated by SGL5213. In contrast, *Turicibacter*, another component of Erysipelotrichaceae, was increased in the RF and further increased in the SGL5213 group. These data suggest that the genus component of Erysipelotrichaceae is related to RF and that SGL5213 affects the composition. Within Bacteroidetes, *Bacteroides* and *S24*‐*7*;*g* (major components of Bacteroidetes), and *Rikenellaceae (unclassified*) were decreased in the RF group, and the reduction was canceled by SGL5213. The increase in Firmicutes genera and decrease in Bacteroidetes mainly changed the F/B ratio in RF, and SGL5213 altered the composition following the reduction of uremic toxins in the RF mice (Figure 3e). In addition, *Bifidobacterium*, a member of the family Bifidobacteriaceae, was significantly increased in the RF group, but the increase was canceled by SGL5213. In contrast, *Corynebacterium* was only increased in the RF + SGL5213 group. Taken together, these data suggest that SGL5213 altered the gut microbiota community in mice with adenine‐induced RF, which may be responsible for the reduction in gut‐derived uremic toxins. The detailed data of taxa at the rank of order, family, and genus are also arranged in Figures S1–S5.

### 3.5 SGL5213 partially inhibit tyrosine phenol‐lyase activity

**FIGURE 7:**
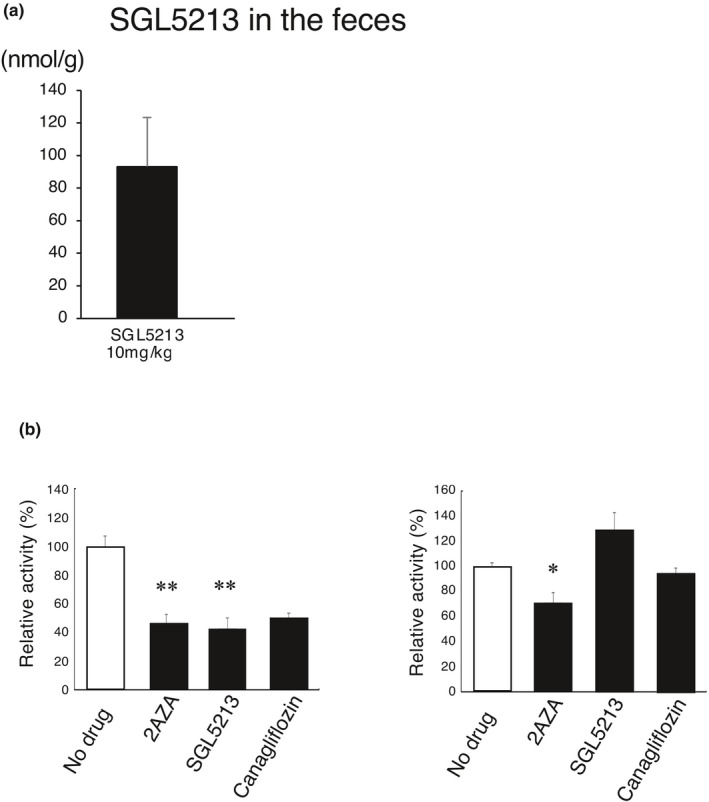
Concentration of SGL5213 in the feces and effect on TPL activity. (a) The concentration of SGL5213 in the feces was measured by LC/MS/MS. Cont: control group (*n* = 8); RF; renal failure group (*n* = 6); RF treated with SGL5213 (10 mg/kg/day) group (*n* = 8–9 each). Data were mean ± SEM. (b) Effect of SGL5213 on TPL activity. One millimolar of the specific TPL inhibitor 2‐aza‐tyrosine, SGL5213, and canagliflozin (SGLT2/1 inhibitor) significantly and comparably inhibited the TPL activity. At 300 μM, 2‐aza‐tyrosine (2‐AZA) still completely and SGL5213 partly inhibited TPL activity. Data were mean ± SEM. Statistical analyses were performed using a Dunnett's test. *p < 0.05 was treated as statistically significant

DKD is a major cause of RF and is in urgent need of a breakthrough in disease management (Pavkov et al., 2012). We reported that PS is a marker and modifiable therapeutic target for DKD patients (Kikuchi et al., 2019). Dietary L‐tyrosine is converted into phenol in the gut by gut bacterial‐specific TPL (EC 4.1.99.2) (Watkins & Phillips, 2001), and absorbed phenol is metabolized into PS in the liver (Kikuchi et al., 2019). The TPL‐specific inhibitors 2‐aza‐tyrosine (Watkins & Phillips, 2001) and L‐meta‐tyrosine (Bertin et al., 2007) reduced plasma PS levels (Kikuchi et al., 2019). In addition, it has been reported that the estimated drug concentrations in the small intestine and colon are higher than the plasma concentration (Maier et al., 2018), and susceptibility to human‐targeted drugs correlates across bacterial species with anti‐commensal activity. Because SGL5213 reduced the PS level in the RF model, we measured the concentration of SGL5213 in the feces. The concentration of SGL5213 in the feces varied from 11.9 to 202 nmol/g (~93.3 μM) (Figure 7a). To further clarify the effect of reducing PS, we examined the effect of SGL5213 on gut microbial‐derived TPL enzyme activity. One mM of the specific TPL inhibitor 2‐aza‐tyrosine (Watkins & Phillips, 2001), SGL5213, and SGL2/1 inhibitor canagliflozin significantly and comparably inhibited TPL activity (Figure 7b). At 300 μM, 2‐aza‐tyrosine was still completely inhibited, but neither SGL5213 nor canagliflozin inhibited TPL activity. These data suggest that the changes in microbiota may be “secondary” to improvement in host homeostasis and reduced uremic toxins.

## 4 DISCUSSION

SGLT2 is predominantly located in the kidney and is responsible for 90% glucose reabsorption in the kidney (Wright et al., 2011), whereas SGLT1 is mainly located in the brush border membrane of the small intestine and plays a critical role in glucose absorption (Gorboulev et al., 2012). These results indicate the renal absorption contribution rate of SGLT1 (10%) and SGLT2 (90%) in the kidney (Wright et al., 2011). SGLT1 is the primary transporter for glucose absorption from digested nutrients in the gastrointestinal tract (Io et al., 2019). SGLT1‐mediated glucose translocation across the BBM is a rate‐limiting step in small intestinal glucose absorption (Gorboulev et al., 2012; Koepsell, 2017). A reduction in glucose absorption in the duodenum and jejunum changes the glucose concentrations in the ileum and colon, which may influence the secretion of gastrointestinal hormones as well as the gut microbiota (Koepsell, 2017). Recently, we reported that canagliflozin, a dual SGLT1/2 inhibitor, reduced the plasma levels of IS and PCS by influencing the intestinal environment in mice with adenine‐induced RF, such as bacterial carbohydrate fermentation (Mishima et al., 2018). Accordingly, we further examined the gut‐specific effect of SGLT1 inhibitor SGL5213, which inhibits SGLT1 in the small intestine. We found that SGL5213 ameliorated renal damage and dysfunction by modifying the gut microbiota community.

Concerning the renoprotective effect of SGL5213, two possible causes are suggested: First, our study revealed that SGL5213 inhibited intestinal glucose absorption and that excess glucose might enhance bacterial carbohydrate fermentation, thereby ameliorating the intestinal microbiota composition in RF mice. We also measured the intestinal and fecal glucose levels (Figure 2a). Although the secretion of glucose into the intestinal fluid was significantly increased in the RF + SGL5213 group compared with the RF group, the fecal glucose level did not change, suggesting that the transit glucose is used in the gut. Indeed, it was anticipated that SGL5213 would reduce the accumulation of gut‐derived uremic toxins by modifying the gut microbiota, which could delay the onset of RF, providing a novel and potential therapeutic tool for CKD patients. Second, colonic transit time is a highly important factor to consider in microbiome and metabolomic studies (Roager et al., 2016). We and other groups reported that several laxatives, lubiprostone (Mishima et al., 2015), linaclotide (Nanto‐Hara et al., 2020), and lactulose (Sueyoshi et al., 2019) modify the gut microbiota and ameliorate CKD progression by suppressing uremic toxin production. Genetic mutations within the SGLT1 gene (*Slc5a1*) in humans are associated with the neonatal onset of severe life‐threatening diarrhea and dehydration due to increased colonic carbohydrates (Al‐Lawama et al., 2019; Lehmann & Hornby, 2016). Oral administration of an SGLT1 selective inhibitor in diabetic rat models also reduced blood glucose, with diarrhea occurring even at a higher dose (Fushimi et al., 2013). In this study, the weight of feces did not change and no obvious diarrhea was observed, suggesting that the renoprotective effect of SGL5213 is not dependent on decreasing colonic transit (Roager et al., 2016).

SGL5213 also reduced the uric acid allantoin levels. Because allantoin is a urate oxidation product (Becker, 1993) and these compounds may increase as a result of the oxidative state in RF, SGL5213 also reduced the renoprotective effect by reducing oxidative stress.

We also found that SGL5213 modulated the gut microbiota composition, characterized by a decreased ratio of Firmicutes to Bacteroidetes and reduced abundance of *Allobaculum* in RF mice. *Allobaculum* is an important functional phylotype of metabolic dysbiosis (Nobel et al., 2015) (Jia et al., 2017). In response to a high‐fat diet (HFD), the B/F proportions reversed and the genus *Allobaculum*, known to be associated with HFD, increased (Nobel et al., 2015). Reduction of *Allobaculum* by SGL5213 can reverse the dysbiotic community. These data suggest that a change in the gut microbial ecosystem by SGL5213 could be an important factor to consider in microbiome and metabolomics studies.

In this study, we found that the F/B ratio in the RF was opposite to that in our previous study (Mishima et al., 2018). It is widely recognized that the intestinal microbiota plays an essential role in health and disease. However, mice bred in the same facility or purchased from a vendor sometimes display differences in the intestinal microbial community (Franklin & Ericsson, 2017). It has also been reported that microbiota differs between commercial breeders and company sourced mice. Diet and other important considerations may determine the model reproducibility (Ericsson & Franklin, 2021) and phenotype (Sadler et al., 2017). Therefore, we tried to obtain mice from the same supplier that produced commercially available laboratory mice and carefully interpreted the results.

Among gut‐derived uremic toxins, SGL5213 significantly decreased the PS (Figure 3d). PS is a metabolite of phenol in humans and is synthesized from dietary L‐tyrosine by gut bacterial TPL (Watkins & Phillips, 2001). TPL inhibition decreases plasma PS levels without inducing host toxicity (Kikuchi et al., 2019). We recently reported that a reduction in PS is a potent therapeutic target for diabetic nephropathy (Kikuchi et al., 2019). Here, we report that SGL5213 reduced the plasma PS level as well as that of glucose, and therefore, could be an ideal drug for diabetic nephropathy. TPL inhibition reduces albuminuria in diabetic mice (Kikuchi et al., 2019). Because it is well known that the estimated drug concentrations in the small intestine and colon are higher than the plasma concentration (Maier et al., 2018), we measured the SGL5213 concentration in the feces. Our data suggest that the effect of SGTl5213 on PS reduction may depend mainly on changes in the gut microbiome community. Recently, it was reported that susceptibility to antibiotics and human‐targeted drugs correlates across bacterial species with anti‐commensal activity. A high concentration of SGLL5213 in feces may result, in part, to a reduction in PS.

Here, we used adenine‐induced CKD mice that crystallized adenine accumulated within the nephron, and the damage was reversible after stopping feeding (Kamijo‐Ikemori et al., 2016; Mishima et al.,, 2018, 2015). To clarify the characteristics of diabetic DKD with Kimmelstiel–Wilson nodules (Conti et al., 2018; Thomas et al., 2015). In mice, there are few models that are suitable for exploring DKD as KK‐Ay/Ta (Ito et al., 2006) or mice lacking eNOS by introducing the Akita mutation in the insulin2 gene (Kikuchi et al., 2019). In addition, the experiment should be longer in length, if possible, extending several months. Furthermore, different long‐span experiments are needed to determine the effect of SGL5213 on DKD.

In conclusion, SGL5213 is an ideal candidate for DKD that promotes the reduction of blood glucose and gut‐derived uremic toxins (especially PS), as well as controlling the bacterial commensal community.

## CONFLICT OF INTEREST

None.

## AUTHOR CONTRIBUTIONS

Conceptualization: Hsin‐Jung Ho, Takaaki Abe. Data curation: Yoshitomi Kanemitsu, Daisuke Saigusa. Formal analysis: Hsin‐Jung Ho. Funding acquisition: Takaaki Abe. Investigation: Hsin‐Jung Ho, Koichi Kikuchi, Daiki Oikawa, Shun Watanabe, Yoshitomi Kanemitsu, Ryota Kujirai, Wakako Ohtsubo, Mariko Ichijo, and Yukako Akiyama. As well as Eikan Mishima, Yoshiaki Ogata, Yoshitsugu Oikawa, Tetsuro Matsuhashi, Takafumi Toyohara, Chitose Suzuki, Takehiro Suzuki, and Takane Katayama. Statistical analysis: Hsin‐Jung Ho, Koichi Kikuchi, and Yuichi Aoki. Project administration: Takaaki Abe. Histological analysis: Yoshiteru Kagawa, Yuji Owada. Tyrosine measurement: Ryota Kujirai. Supervision: Toru Nakayama, Nariyasu Mano, Yoshihisa Tomioka, and Takaaki Abe. Writing—original draft: Hsin‐Jung Ho, Takaaki Abe. Writing—review and editing: Hsin‐Jung Ho, Koichi Kikuchi, Takaaki Abe.

## Supporting information



Supplementary Material
