# Supplementary material for: SGLT‐1‐specific inhibition ameliorates renal failure and alters the gut microbial community in mice with adenine‐induced renal failure
Source: Physiol Rep. 2021 Dec 18;9(24):e15092. doi: 10.14814/phy2.15092 (PMC8683788; doi:10.14814/phy2.15092)
Supplement: Supplementary file 1 — Supplementary Material [file PHY2-9-e15092-s001.pdf]

Supplementary Data set

Title: SGLT-1-specific inhibition ameliorates renal failure and alters the gut microbial community in mice with adenine-induced renal failure

Hsin-Jung Ho*, Koichi Kikuchi*, Daiki Oikawa*,
Shun Watanabe*,Yoshitomi Kanemitsu, Daisuke Saigusa,
Ryota Kujirai, Wakako Ohtsubo, Mariko Ichijo,
Yukako Akiyama, Eikan Mishima, Yoshitsugu Oikawa, Tetsuro Matsuhashi, Takafumi Toyohara, Chitose Suzuki, Takehiro Suzuki, Nariyasu Mano, Yoshiteru Kagawa, Yuji Ohwada, Takane Katayama, Toru Nakayama, Yoshihisa Tomioka and Takaaki Abe

Supplementary Figure 1

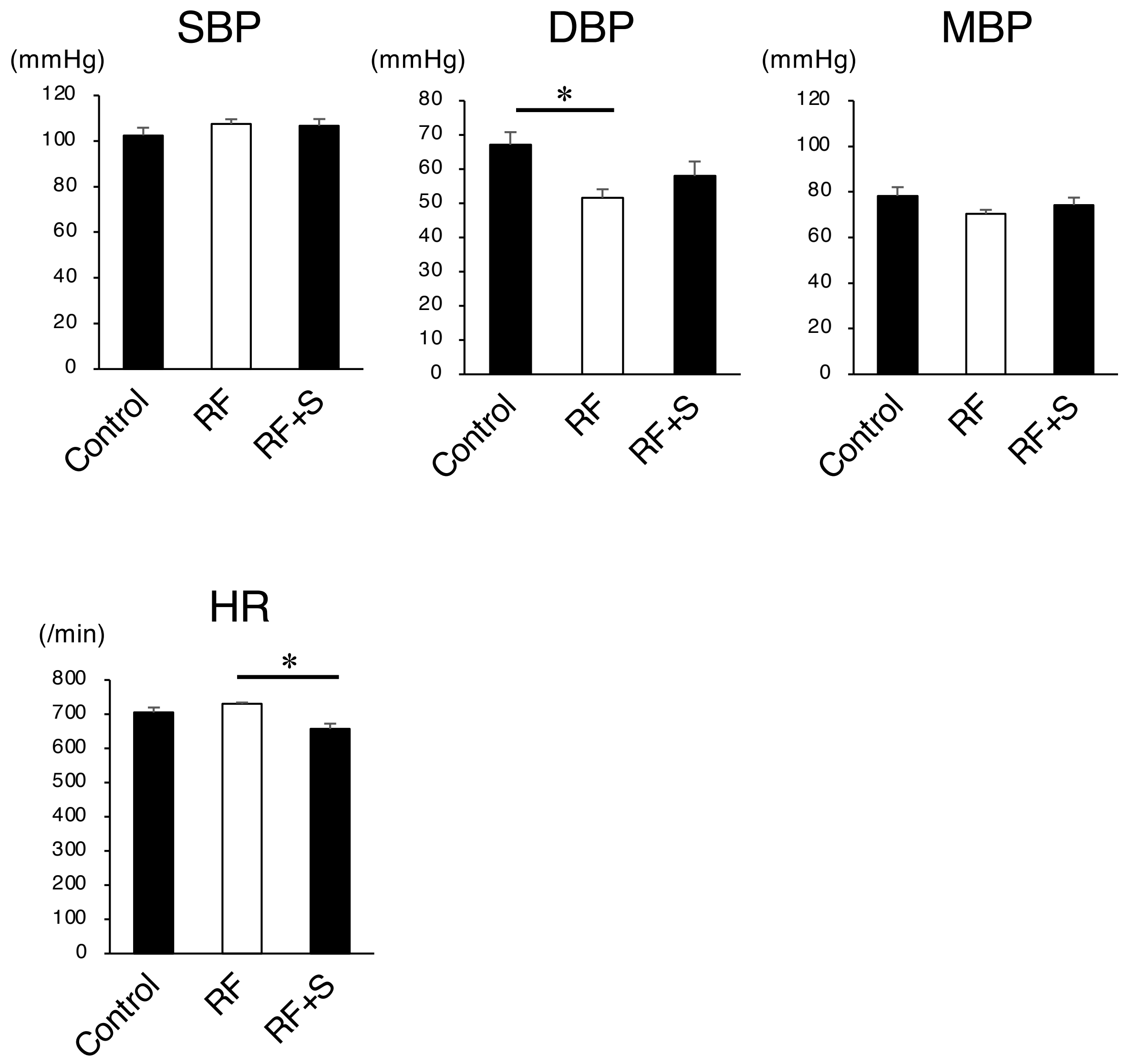

Blood pressure and heart rate were measured by MK-2000ST (Muromachi, Japan).
Control (n=6), RF: renal failure (n=6), RF + S: renal failure with SGL5213 treatment (n=6)
SBP: systolic blood pressure, DBP: diastolic blood pressure, MBP: mean blood pressure
HR: heart rate.

Data were shown by mean ± SEM.
*p<0.05, Ordinary one-way ANOVA followed by Dunnett's multiple comparison test vs RF.

Supplementary Figure 2

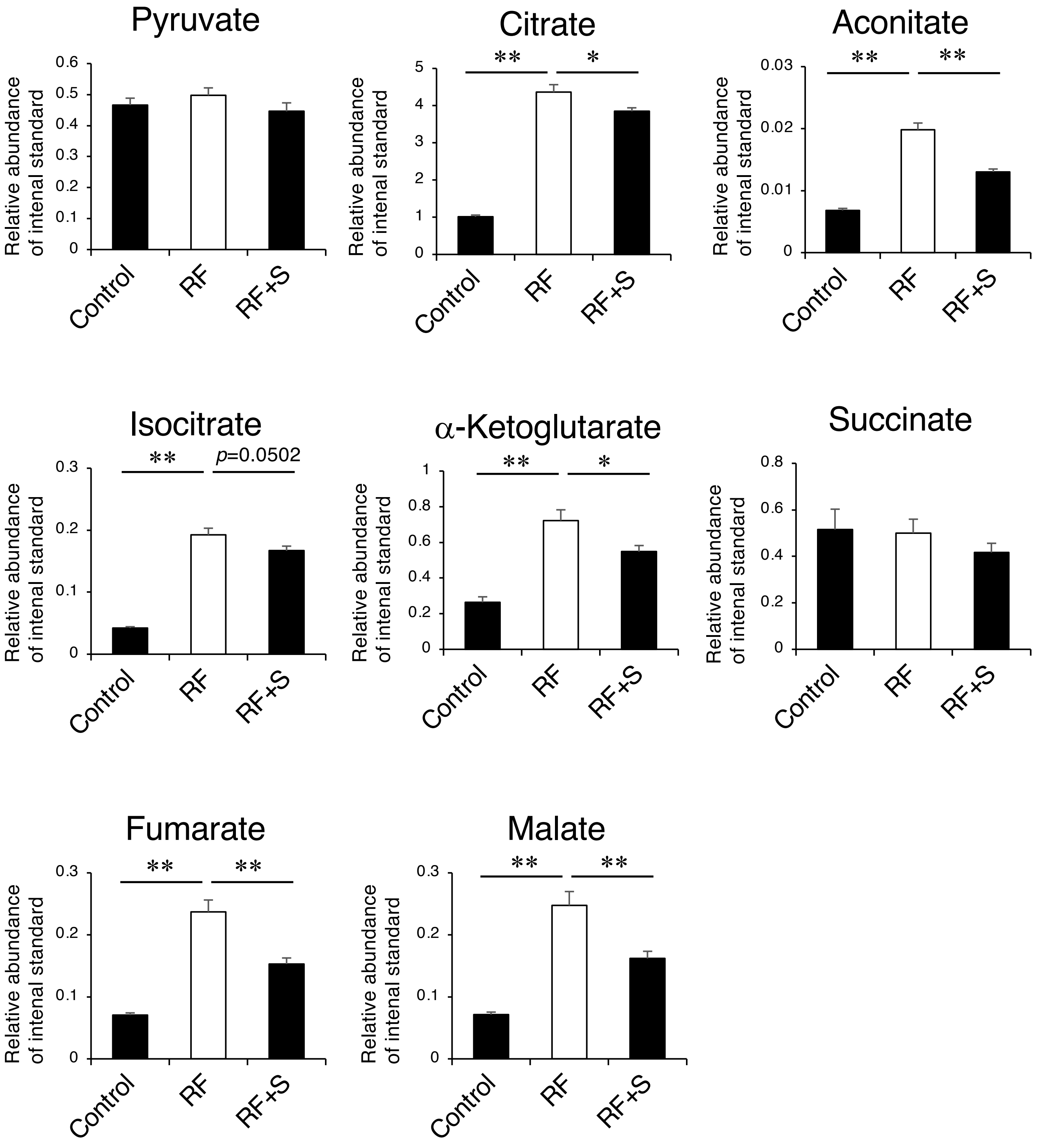

Internal standard: 2-Isopropylmalic acid-3TMS
Mean ± SEM. *p<0.05, **p<0.01 Ordinary one-way ANOVA followed by Dunnett's multiple comparison test vs RF.

Supplementary Figure 3

phylum

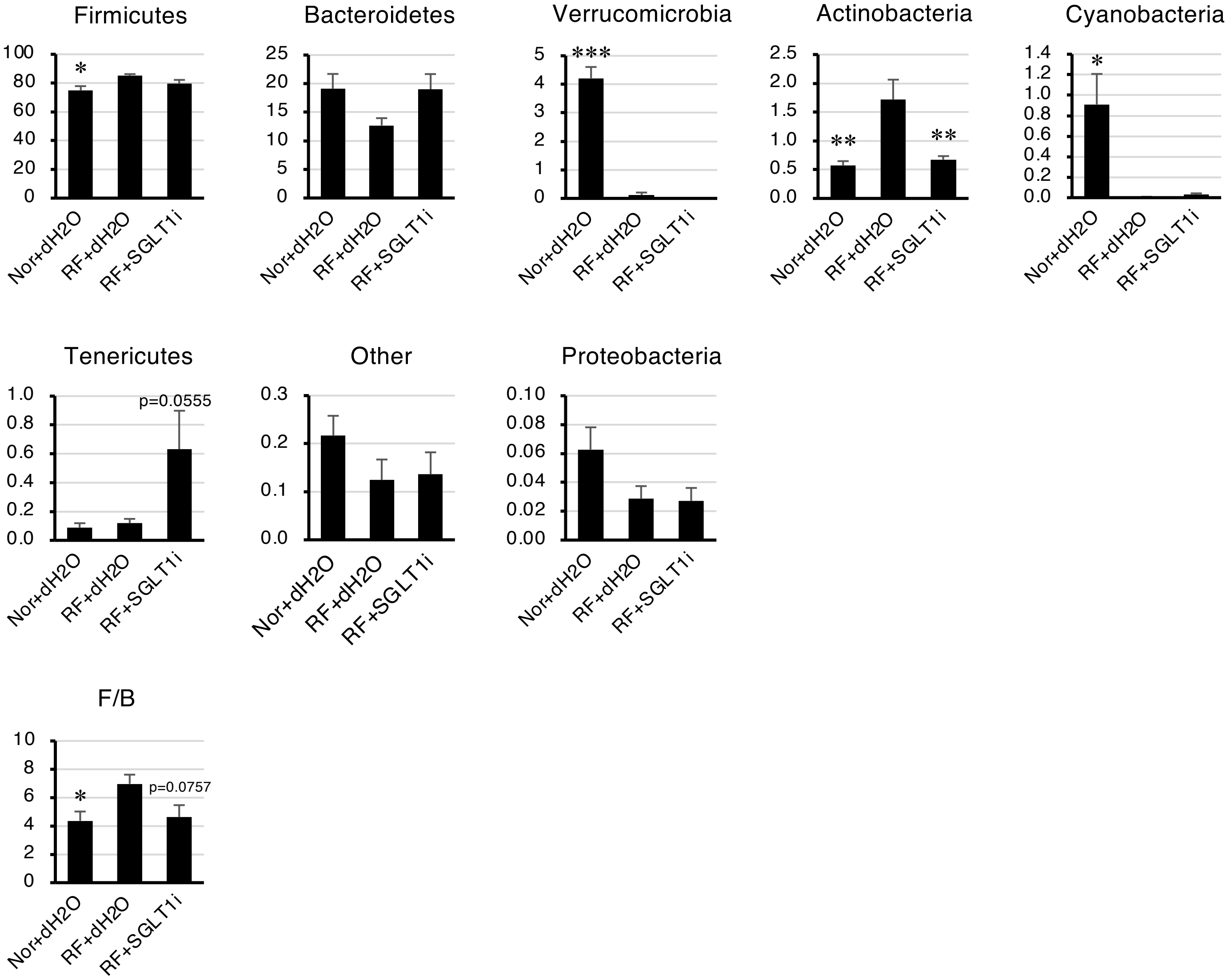

Supplementary Figure 4

class

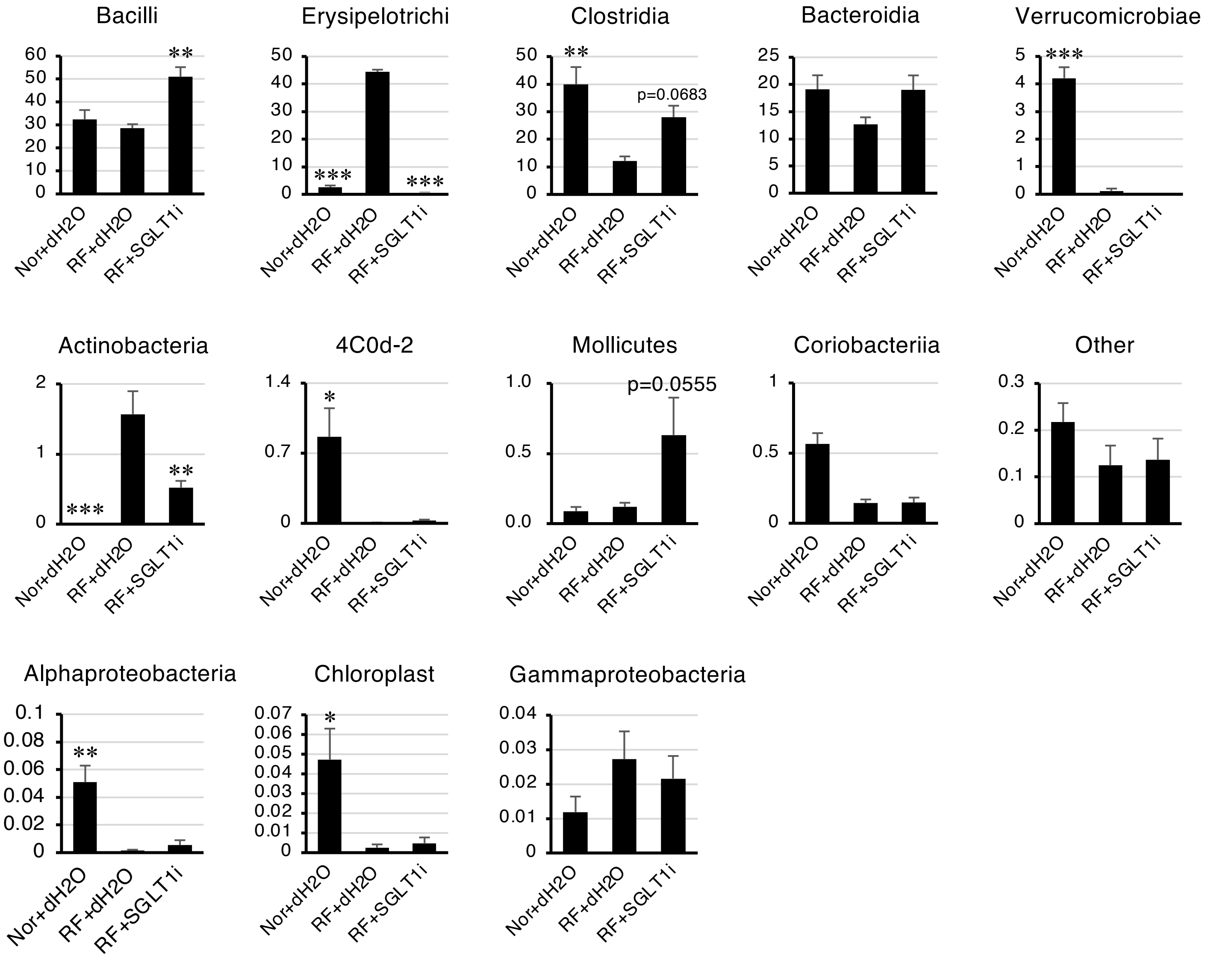

Supplementary Figure 5
order

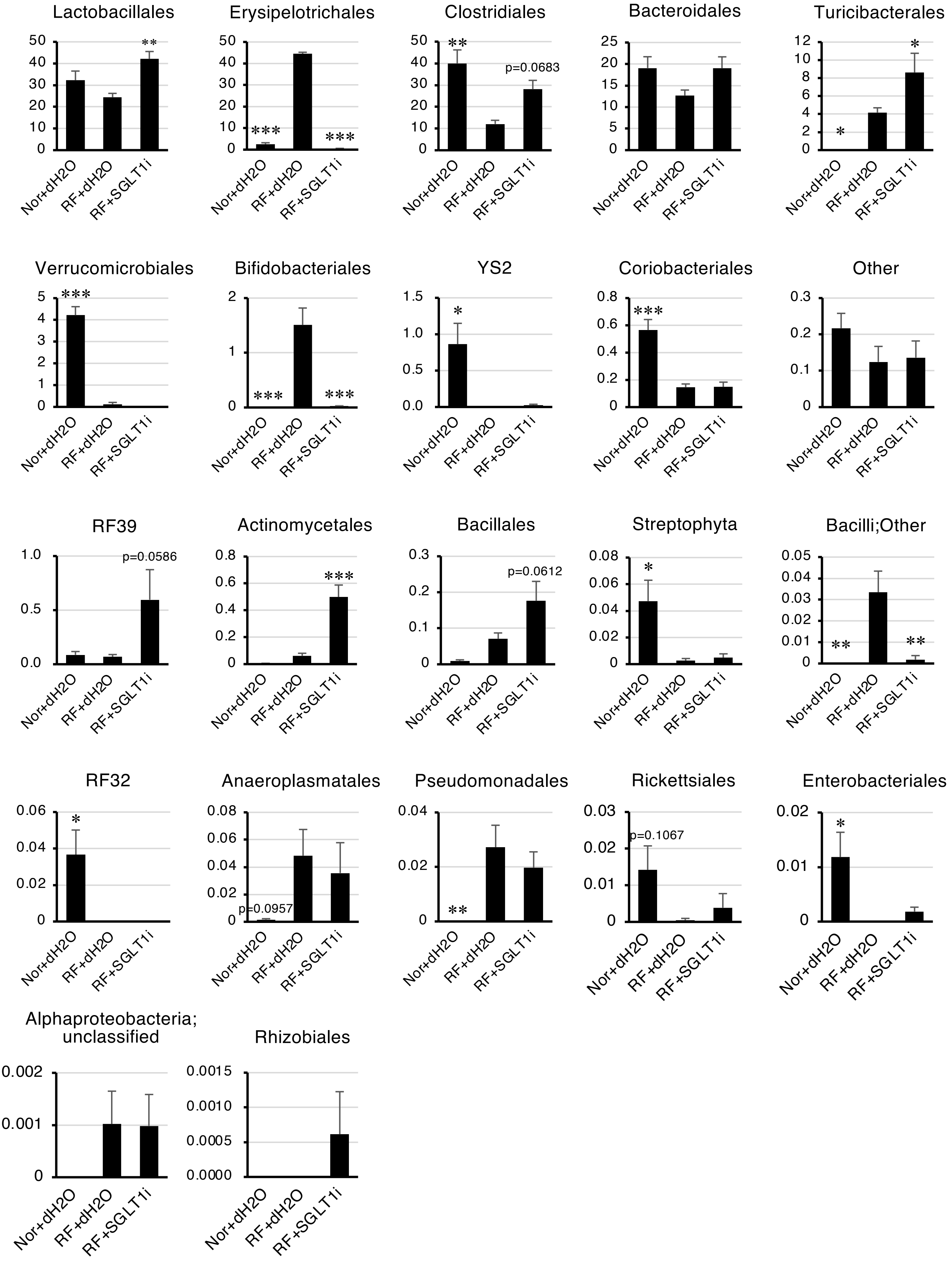

Supplementary Figure 6-1

family

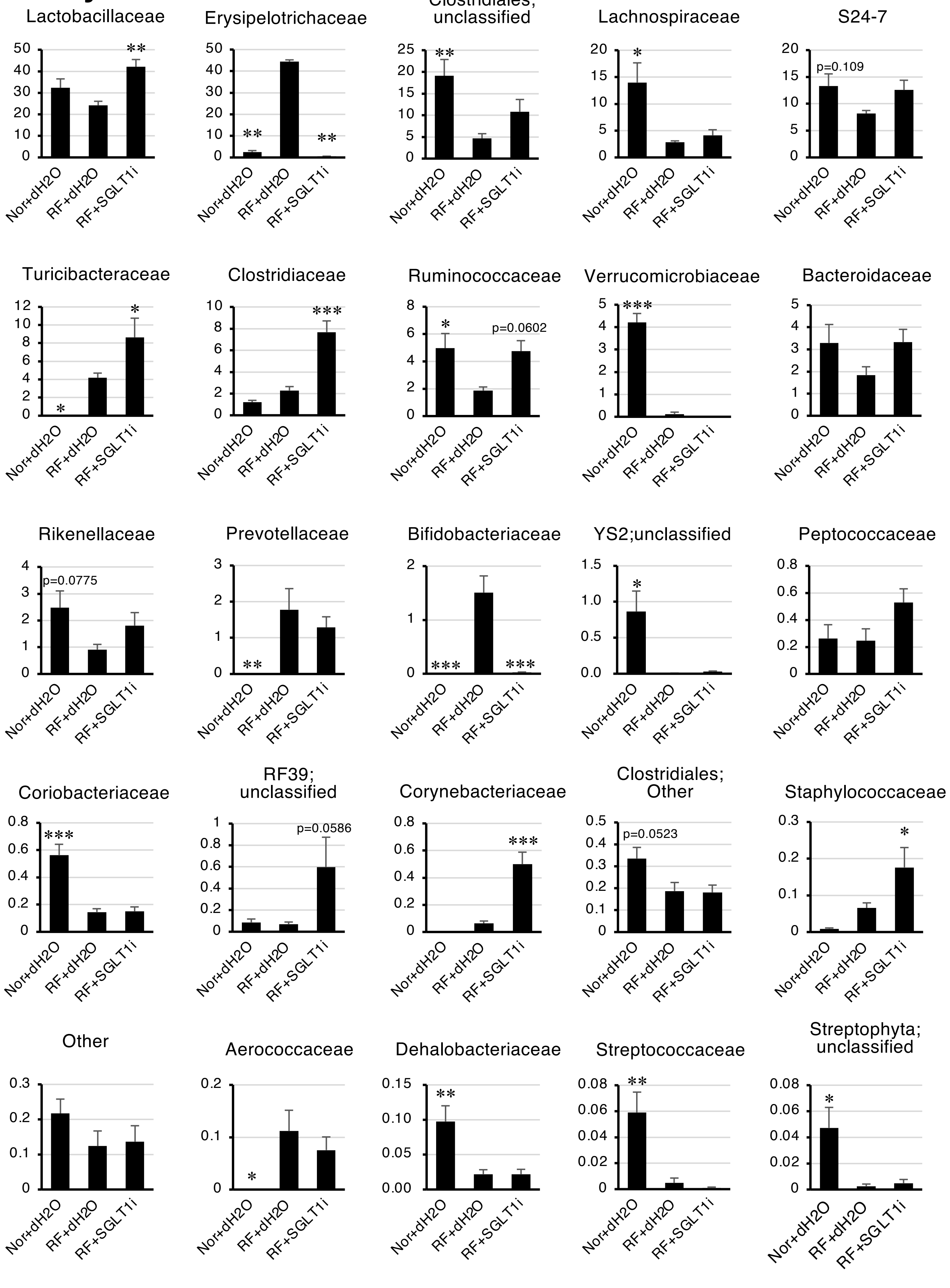

Supplementary Figure 6-2
family

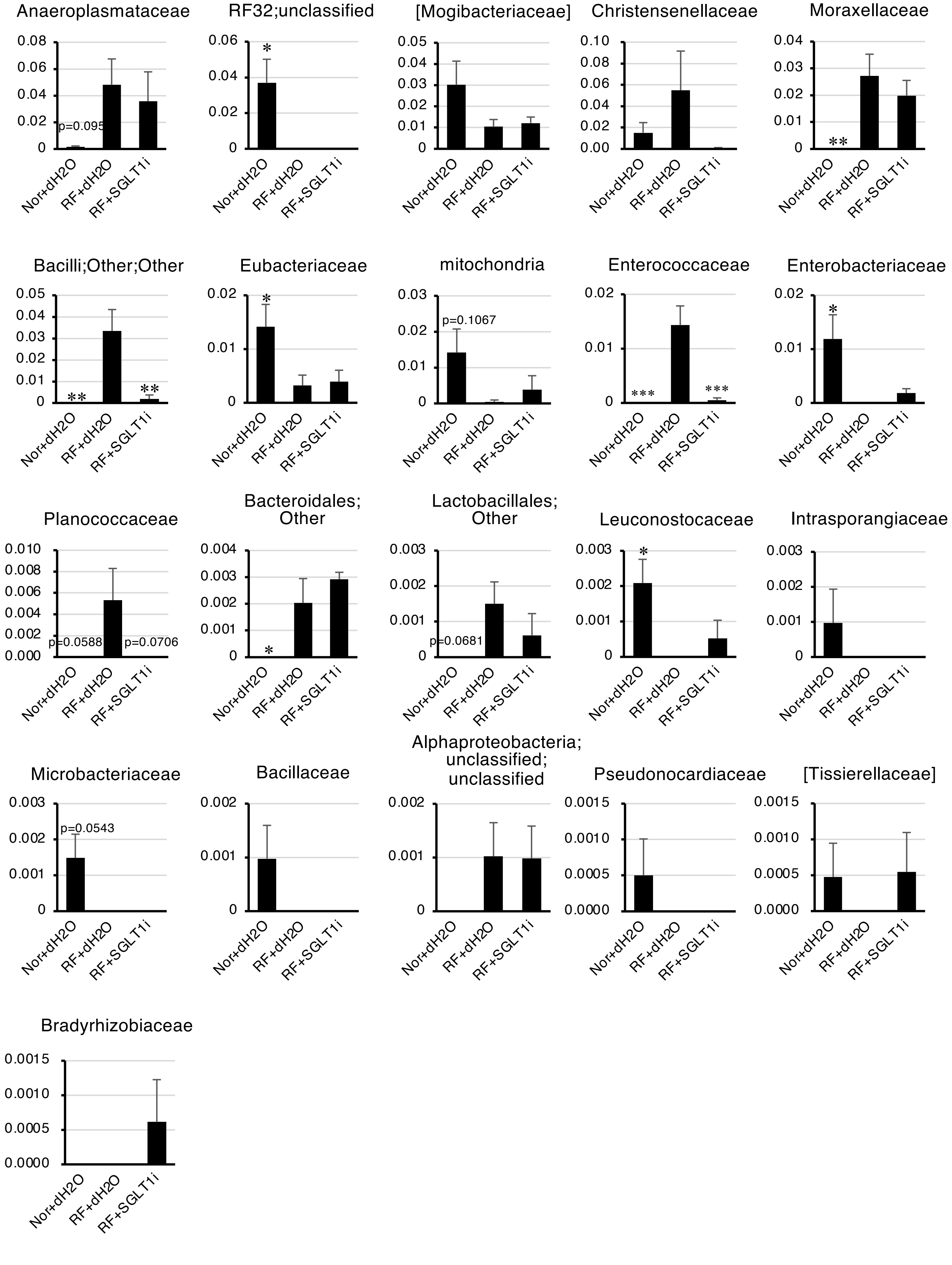

Supplementary Figure 7-1
genus

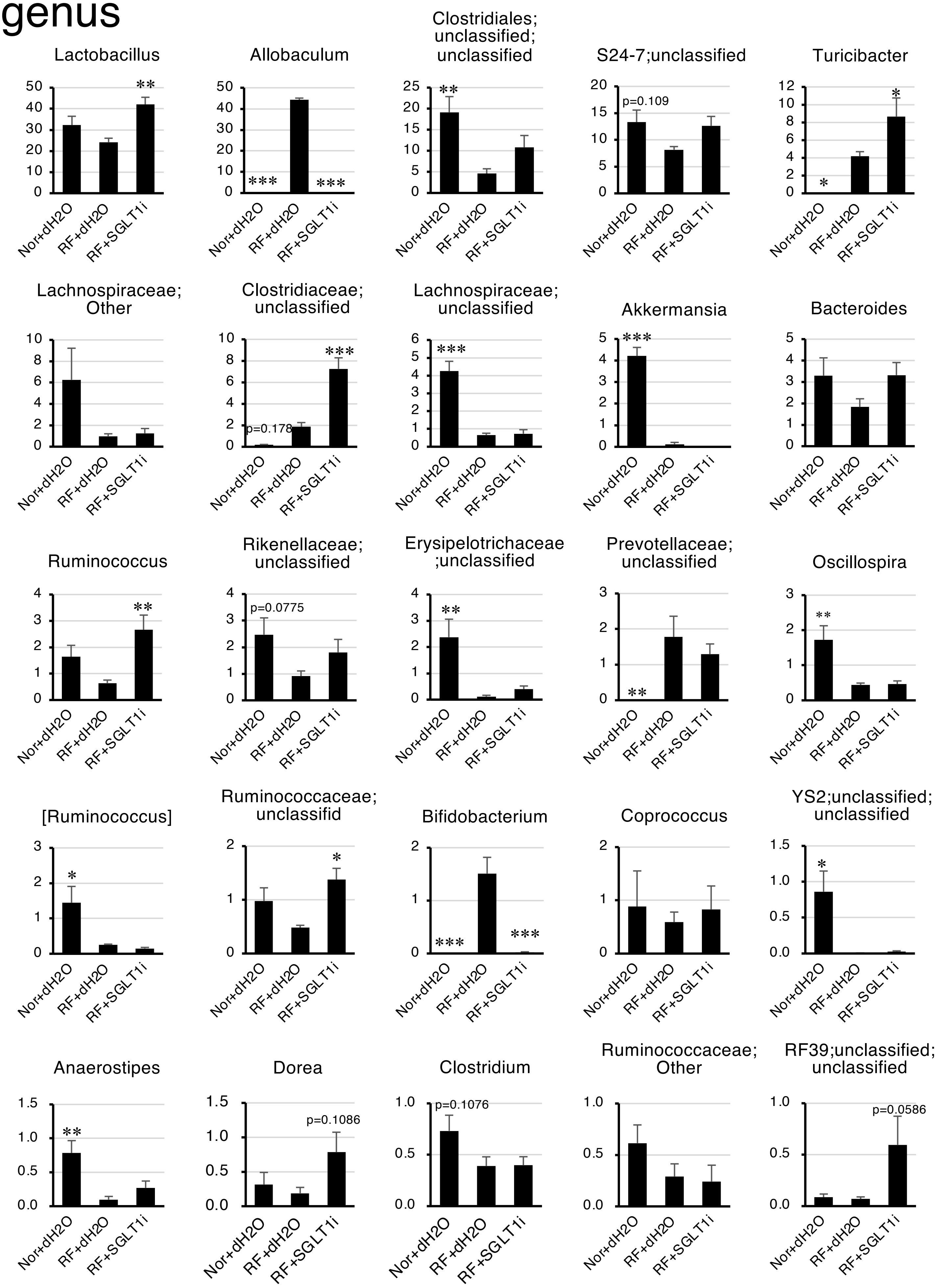

Supplementary Figure 7-2
genus

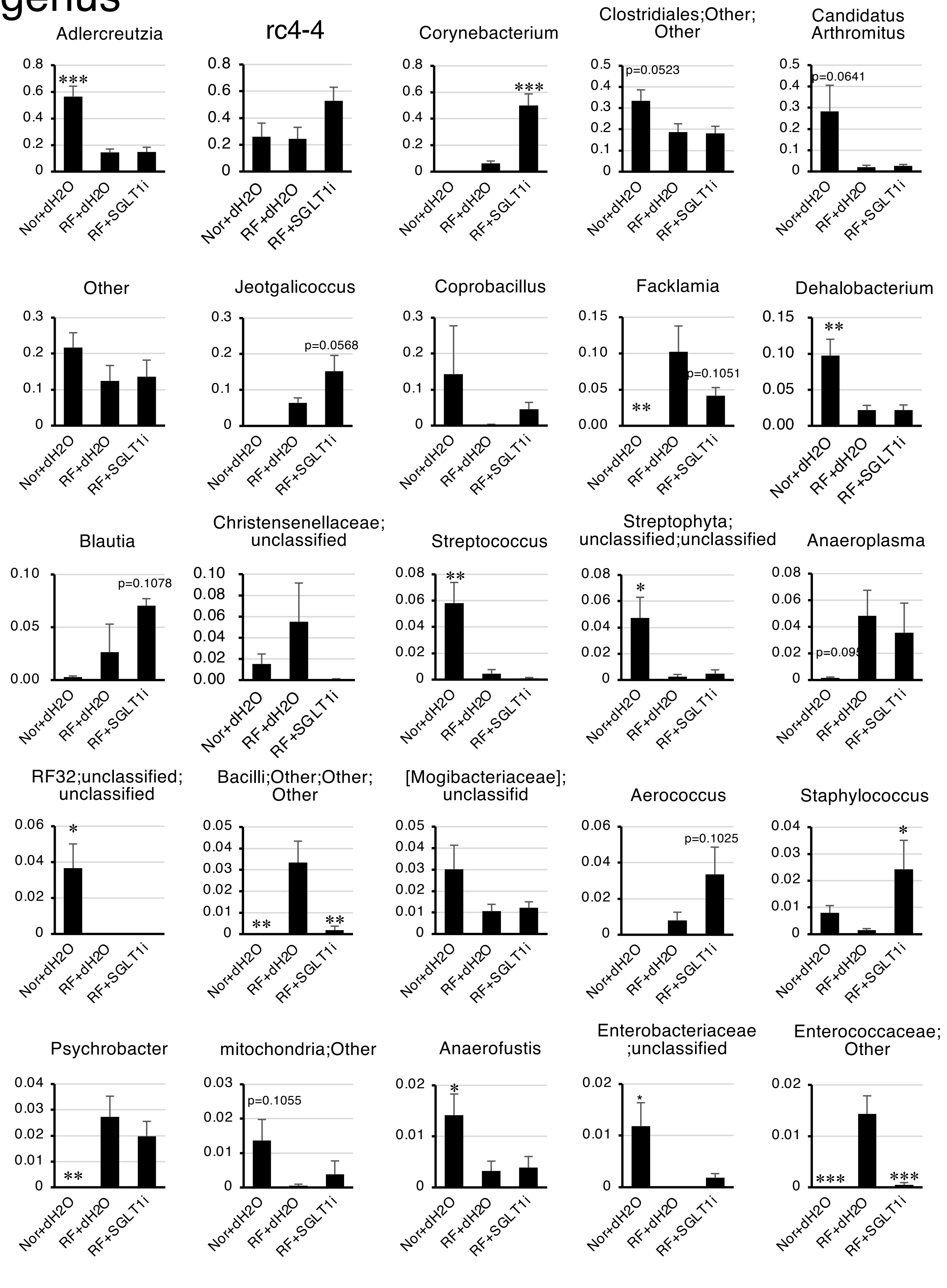

Supplementary Figure 7-3
genus

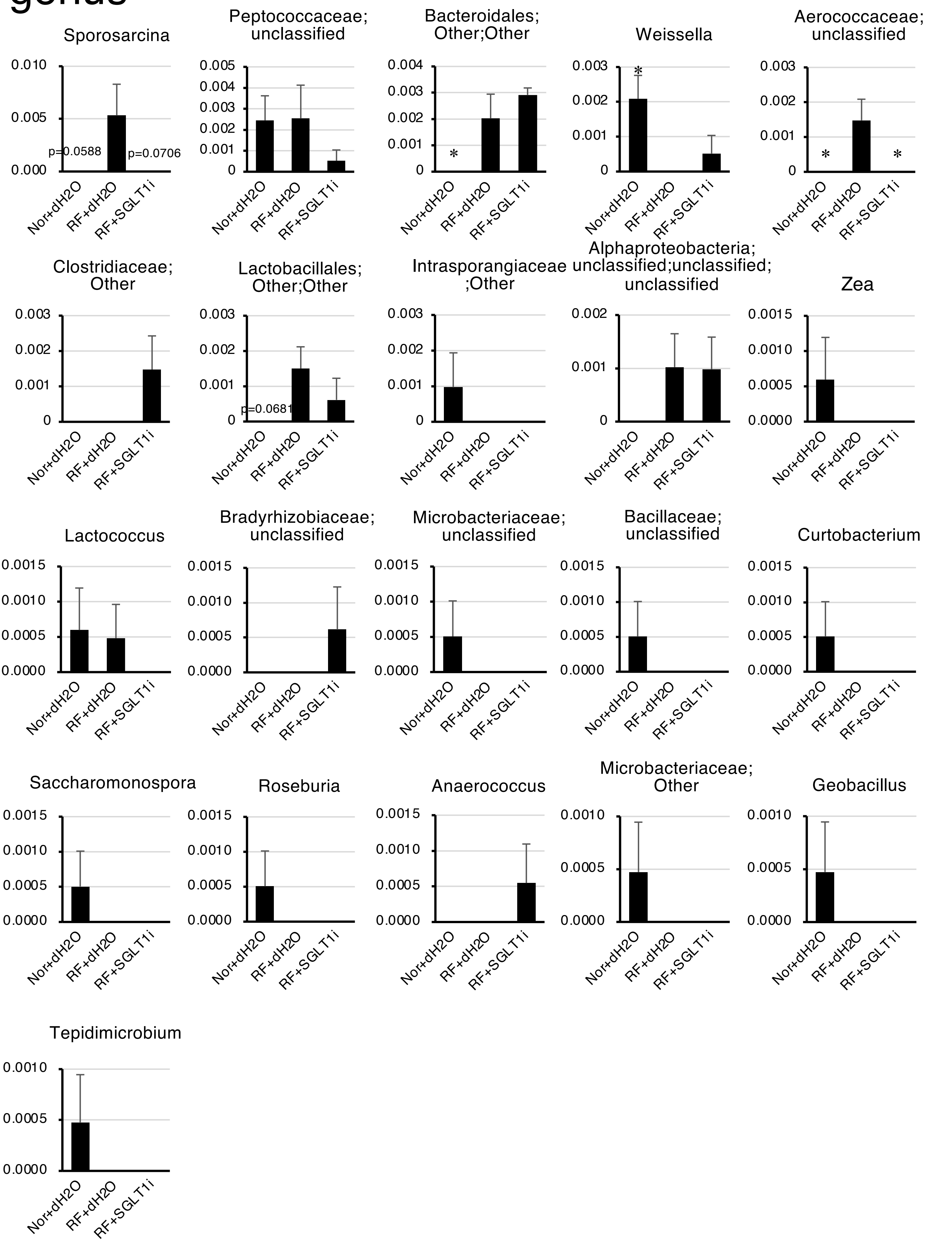

Supplementary Table 1

Taqman Gene Expression Assays	
Ccl2	Mm00441242_m1
Emr1	Mm00802529_m1
Tgfb1	Mm01178820_m1
Col1a1	Mm00801666_g1
Acta2	Mm00725412_s1
Cd68	Mm03047343_m1

Supplementary Table 1
List of primers used in the PCR analysis. Primers were purchased from Applied Biosystems. The sequences of the primers and probes are certificated by the company, but not open by company policy.
